# The catalytic mechanism of the mitochondrial methylenetetrahydrofolate dehydrogenase/cyclohydrolase (MTHFD2)

**DOI:** 10.1371/journal.pcbi.1010140

**Published:** 2022-05-25

**Authors:** Li Na Zhao, Philipp Kaldis

**Affiliations:** 1 Department of Clinical Sciences, Lund University, Malmö, Sweden; 2 Lund University Diabetes Centre, Lund University, Malmö, Sweden; University of Maryland School of Pharmacy, UNITED STATES

## Abstract

Methylenetetrahydrofolate dehydrogenase/cyclohydrolase (MTHFD2) is a new drug target that is expressed in cancer cells but not in normal adult cells, which provides an Achilles heel to selectively kill cancer cells. Despite the availability of crystal structures of MTHFD2 in the inhibitor- and cofactor-bound forms, key information is missing due to technical limitations, including (a) the location of absolutely required Mg^2+^ ion, and (b) the substrate-bound form of MTHFD2. Using computational modeling and simulations, we propose that two magnesium ions are present at the active site whereby (i) Arg233, Asp225, and two water molecules coordinate $Mg_{A}^{2\text{+}}$, while $Mg_{A}^{2\text{+}}$ together with Arg233 stabilize the inorganic phosphate (P_*i*_); (ii) Asp168 and three water molecules coordinate $Mg_{B}^{2\text{+}}$, and $Mg_{B}^{2\text{+}}$ further stabilizes P_*i*_ by forming a hydrogen bond with two oxygens of P_*i*_; (iii) Arg201 directly coordinates the P_*i*_; and (iv) through three water-mediated interactions, Asp168 contributes to the positioning and stabilization of $Mg_{A}^{2\text{+}}$, $Mg_{B}^{2\text{+}}$ and P_*i*_. Our computational study at the empirical valence bond level allowed us also to elucidate the detailed reaction mechanisms. We found that the dehydrogenase activity features a proton-coupled electron transfer with charge redistribution connected to the reorganization of the surrounding water molecules which further facilitates the subsequent cyclohydrolase activity. The cyclohydrolase activity then drives the hydration of the imidazoline ring and the ring opening in a concerted way. Furthermore, we have uncovered that two key residues, Ser197/Arg233, are important factors in determining the cofactor (NADP^+^/NAD^+^) preference of the dehydrogenase activity. Our work sheds new light on the structural and kinetic framework of MTHFD2, which will be helpful to design small molecule inhibitors that can be used for cancer treatment.

## Author summary

MTHFD2 is a newly-discovered therapeutically important enzyme. It is expressed at high levels in acute myeloid leukemia, pancreatic cancer, colorectal cancer, glioma, and renal cell carcinoma, and is associated with poor survival of patients. MTHFD2 is highly expressed in cancer cells but not in healthy cells. Targeting MTHFD2 has become an important strategy in eradicating cancer cells without damaging normal cells. However, there is key information missing due to technical limitations, including (a) the location of the absolutely required Mg^2+^ ion, (b) the substrate-bound form of MTHFD2, and (c) the catalytic mechanism of MTHFD2. In order to better target MTHFD2, it is important to gain in-depth understanding about its structure and the molecular basis of mechanism of action. In our work, we have reconstructed the structural information and investigated the mechanism of MTHFD2 at the molecular level. We found that the dehydrogenase activity features (a) proton-coupled electron transfer, and (b) a charge redistribution, which promotes the subsequent cyclohydrolase activity. Furthermore, we have observed that a second Mg^2+^ enhances binding of a cofactor, and Ser197/Arg233 defines the cofactor NADP^+^/NAD^+^ selectivity.

## Introduction

Chemotherapeutic drugs that aim to kill cancer cells unavoidably also impact healthy normal cells causing undesirable side effects. Methylenetetrahydrofolate dehydrogenase 2 (MTHFD2) has emerged as new drug target due to (a) its expression only during embryonic development but not in adult tissue, and (b) its amplification in cancer cells [1–5]. Overexpression of MTHFD2 provides cancer cells with the necessary building blocks for nucleotide (purine and pyrimidine) biosynthesis during rapid proliferation [2]. Targeting MTHFD2 is a novel anticancer therapeutic approach to kill tumor cells without damaging healthy cells [2, 5–7].

**Fig 1:**
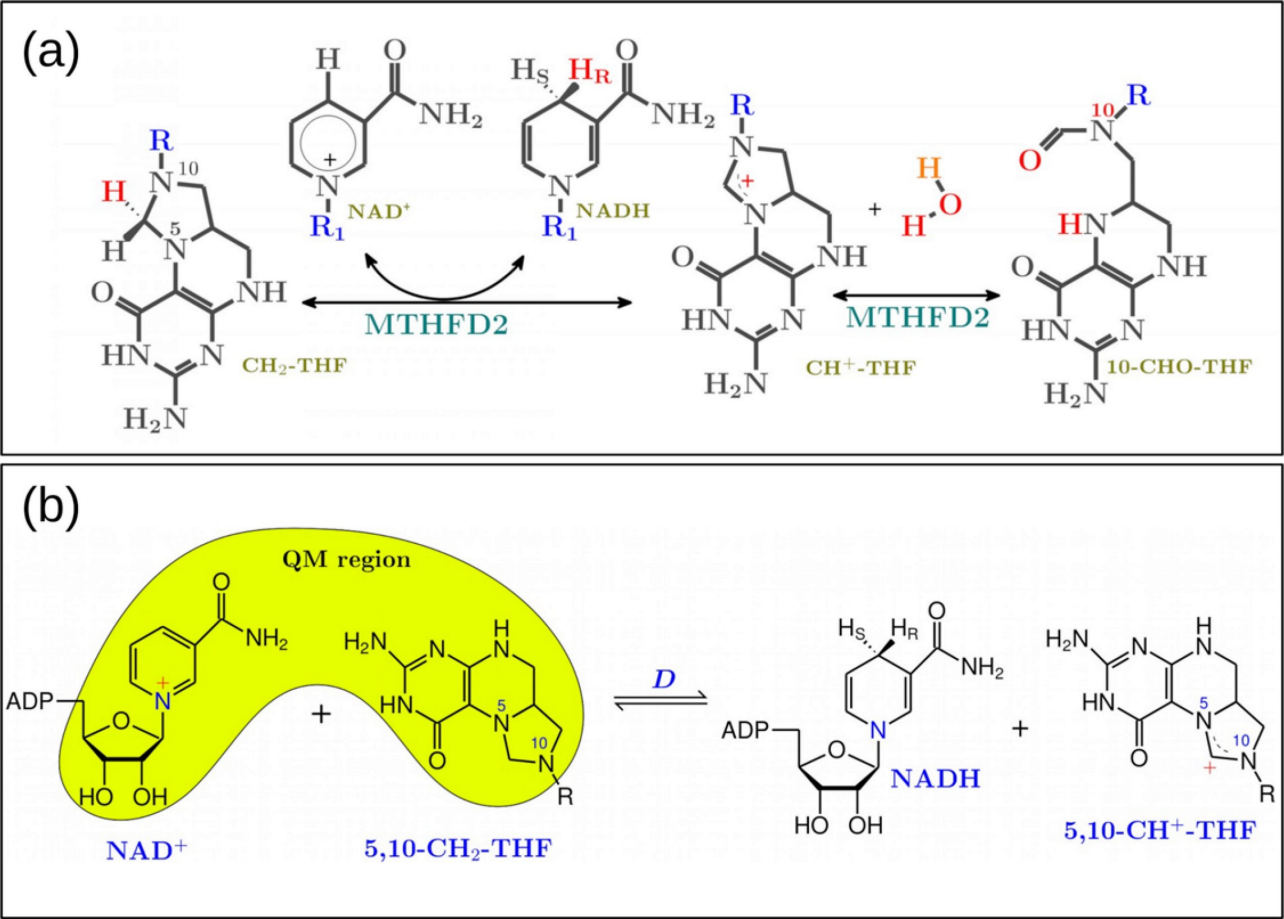
(a) The MTHFD2 reaction scheme. (b) The dehydrogenase (**D**) activity. Note that the QM region indicates the EVB atoms used for our empirical valence bond simulation of the **D** activity. R = p-aminobenzoyl-glutamate, R_1_ = Ribose-ADP.

MTHFD2 carries out both methylenetetrahydrofolate dehydrogenase (***D***) and cyclohydrolase (***C***) activities that are derived from its trifunctional precursor methylenetetrahydrofolate dehydrogenase, cyclohydrolase and formyltetrahydrofolate synthetase 1 (MTHFD1) through the loss of the *C-*terminal synthetase domain and a novel adaptation to NAD^+^ rather than NADP^+^ as cofactor for the dehydrogenase activity (Fig 1) [8]. Although the 5,10-methylenetetrahydrofolate (5,10-CH_2_-THF) dehydrogenase activity is well recognized as NAD^+^-dependent with an absolute requirement for Mg^2+^ and inorganic phosphate (P_*i*_) in NAD^+^ binding, the two ions have no effect on THF binding [8]. Neither the cofactor (NADP^+^/NAD^+^) nor the ions are required for the MTHFD2 cyclohydrolase activity [8]. Other enzymes with dehydrogenase activities such as its cytosolic counterparts MTHFD1, utilize NADP^+^ as cofactor whereas the mitochondrial isozyme MTHFD2L, can use either NAD^+^ (when using NAD^+^ it also requires both Mg^2+^ and phosphate) or NADP^+^ for dehydrogenase activity [9]. Recent studies suggest that MTHFD2 can achieve higher catalytic efficiency when using NAD^+^ rather than NADP^+^ [10]. When using 5,10-CH_2_-H_4_PteGlu_1_ and 5,10-CH_2_-H_4_PteGlu_5_ as substrates, the NAD^+^-dependent dehydrogenase activity is 8.5 and 2.4 times higher than its NADP^+^-dependent activity, respectively [10]. The increase in maximal activity when using NAD^+^ as cofactor rather than NADP^+^ is important because it increases the production of formyl-THF in mitochondria [11], which meets the high demand for glycine and purine during proliferation in cancer and embryonic cells [2]. However, which molecular features of MTHFD2 contribute to the cofactor specificity change is currently unknown.

The monomeric MTHFD2 consists of D and C domains responsible for the ***D*** and ***C*** activities. The D/C domains of MTHFD2 form a cleft, which is composed of two *α*/*β* strands that assemble together. The walls of the cleft feature highly conserved residues [12]. NAD^+^ and P_*i*_ are bound along one wall, while the substrate is bound at the interface between the two domains. MTHFD2 functions as a homodimer with homodimerization occurring by antiparallel interaction of the two NAD^+^-binding domains [12,13].

Although magnesium has long been recognized as an essential metal cation for both the NAD^+^- and NADP^+^-dependent dehydrogenase activities of MTHFD2, it has been difficult to crystallize the MTHFD2 complex together with Mg^2+^ [11,14]. The monomeric crystal structure of MTHFD2 (5TC4) was solved at 1.89 Å with NAD^+^, P_*i*_, and the inhibitor LY345899. Subsequently, homodimeric MTHFD2 was co-crystallized with three inhibitors (Compound 1, 6JID; Compound 18, 6KG2; and DS44960156, 6JIB) in the presence of cofactors (NAD^+^ and P_*i*_) at 2.25Å, respectively. However, the absolutely required Mg^2+^ is missing in all these 4 structures. Therefore, the location of Mg^2+^ in the MTHFD2 is not known.

P_*i*_ has been observed to occupy a position next to the 2′-hydroxyl of NAD^+^, which mimics the space that would otherwise be occupied by the 2′-phosphate of NADP^+^ [8]. Previous work has identified two residues, Arg201 and Arg233, that are important for the binding of P_*i*_ or the 2’-phosphate of NADP^+^ [8]. It is not known whether Mg^2+^ is directly involved in the P_*i*_ binding but the high Lewis acidity of Mg^2+^ is capable of stabilizing a phosphate anion. Characterization of MTHFD2 mutations at Asp168 and Asp225 indicate that both residues play a primary and direct role in assisting the binding and orientation of the Mg^2+^ ion in the cofactor binding site [8].

Since MTHFD2 functions as dimer and no crystal structure of MTHFD2 with both inorganic phosphate and Mg^2+^ has been obtained, we have reconstructed the model of the MTHFD2 homodimer complex based on available X-ray structures, site-directed mutagenesis and empirical valence bond (EVB) studies, and have quantum chemically located the Mg^2+^ binding sites.

To probe the energetics and mechanism of the initial proton-coupled electron transfer (PCET) process between NAD^+^ and 5,10-CH_2_-THF, and how this connects to the subsequent cyclohydrolase reaction, we have combined density functional theory (DFT) calculations with classical molecular dynamics (MD) simulations and hybrid quantum mechanics/molecular mechanics (QM/MM) calculations using the empirical valence bond approach. We propose a putative mechanism for the opening of the imidazoline ring after the PCET reaction and discuss its possible implications.

## Materials and methods

### MTHFD2 structure construction

**Fig 2:**
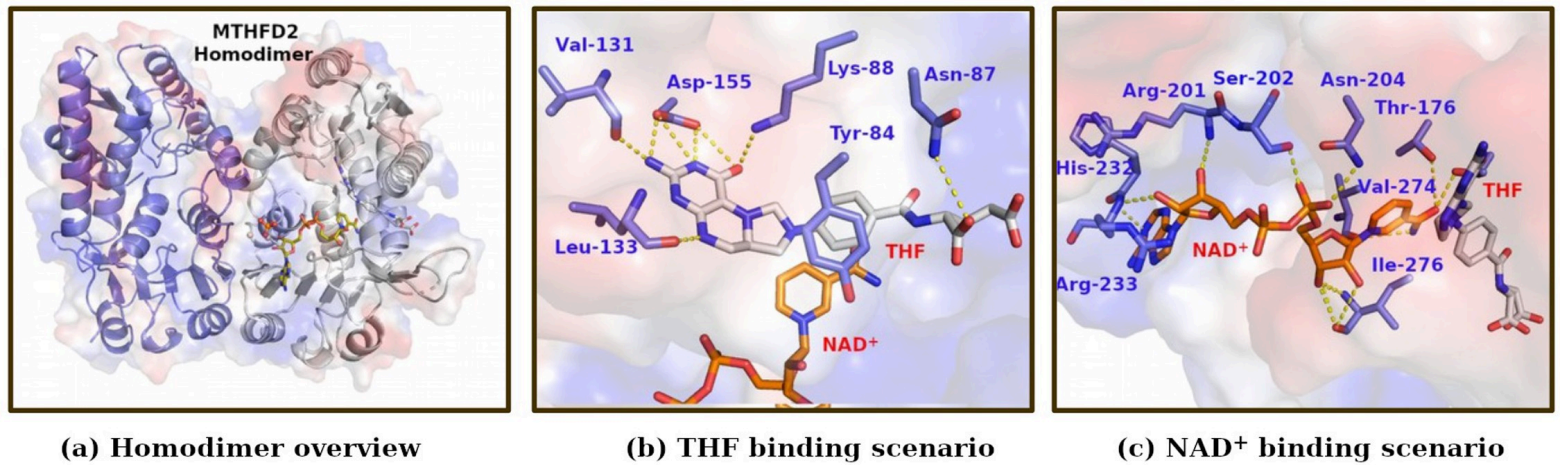
Overview of modeled MTHFD2 system, with THF and NAD^+^ binding pockets. (a) Homodimer overview (b) THF binding scenario (c) NAD^+^ binding scenario.

The initial structure for MTHFD2 was taken from Protein Data Bank (ID: 6KG2). 50 models were generated by "loopmodel" class within MODELLER 9.23 to model and refine the missing loop from residues 281 to 285 [15]. [6R]-5,10-methylene-THF (endogenous one-carbon donor) was built from the ZINC database (ZINC4228244; https://zinc15.docking.org/substances/ZINC000004228244/; note that we have changed the D-glutamate moiety into its native L-glutamate in our simulation study; see S2 and S3 Figs). Dock3.7 was used to dock 5,10-CH_2_-THF into all above 50 models. The conformations from the top ranked pose, where the reactive C atoms from 5,10-CH_2_-THF and NAD^+^ are within a catalytic feasible distance (4.5 Å) and in catalytically competent orientation, were selected for further evaluation (7 in total). In addition, we have added the absolutely required Mg^2+^ at the inorganic phosphate binding site after systematically examining the Mg^2+^ binding pockets in all known crystal structures and integrated this with the mutagenesis studies of MTHFD2 (the details are given in S1 Text). The constructed MTHFD2 structure is shown in Fig 2. Key interactions between THF and MTHFD2 include (see Fig 2B): (i) the backbone of Val131 and Leu133, and the side chain of Asp155, form hydrogen bonds with the Pterin group of THF; (ii) Tyr84, *para-*aminobenzoic acid of THF, and the ring of nicotinamide of NAD^+^ form π–π stackings; and (iii) Asn87 forms a hydrogen bond with the glutamate moiety of THF. These interactions of THF with MTHFD2 recaptures the interaction scenario of inhibitor L34 in the 5TC4 crystal structure. The key interactions between NAD^+^ and MTHFD2 include (see Fig 2C): (i) the backbone of His232 and Arg233 form hydrogen bonds with the adenine group of NAD^+^; (ii) the backbone of Arg201 and Ile276, and side chain of Ser202 and Asn204 form hydrogen bonds with the ribose and phosphates moieties of NAD^+^; and (iii) the side chain of Thr176, and the backbone of Val274 form hydrogen bonds with the nicotinamide group of NAD^+^.

### EVB Simulations

We have carried out density functional theory (DFT) calculations with a continuum solvent model to optimize the geometries of the reactant, transition state and product. The Mulliken charges for our EVB atoms are obtained using the B3LYP method with the 6-31g(d) basis set. In addition, we have used the B3LYP function with larger basis sets cc-pVTZ to obtain a kinetic description of the proton transfer from 5,10-CH_2_-THF to NAD^+^ with more accurate energies. The results from the DFT calculation (the active site model consisted of 38 atoms) were then used to calibrate empirical valence bond models for the proton transfer step. The two Mg^2+^ binding models were optimized in the MTHFD2·NAD^+^·P_*i*_·Mg^2+^·THF complex using constraint_pair within the Molarix-XG package to constrain by the force constant of 10.0 *k*_*cal*_ *mol*^−1^ and around the distance that represents the configuration of the optimized Mg^2+^ configuration. The active site region along with the MTHFD2·NAD^+^·P_*i*_·Mg^2+^·THF complexes were immersed in a 32Å sphere of water molecules using the surface-constraint all-atom solvent (SCAAS) boundary condition [16]. A 2Å layer of Langevin dipoles was applied outside of this 32Å region, followed by a bulk continuum. Atoms beyond the sphere were fixed at their initial positions and no electrostatic interactions from outside of the sphere were considered. The long-range electrostatics was treated with the local reaction field (LRF) method. The geometric center of the EVB reacting atoms was set as the center of the simulation sphere. It is challenging to obtain the accurate pK_a_ of ionizable groups in proteins since it crucially depends on the local environment and varies from region to region. The development of a semi-microscopic version of the protein dipoles Langevin dipoles (PDLD) model has overcome the accuracy issue and provides a consistent way by taking into account the dynamics of charges during structural reorganization [17]. The Monte Carlo proton transfer (MCPT) algorithm was used to optimize the charge distribution of all ionizable residues. MCPT simulates the proton transfer between charged residues in which the charge distribution is updated and evaluated with Monte Carlo approaches to identify the optimal charge distribution. Hence, the protonation state for the ionizable residues is determined by calculating MCPT. The EVB atoms are given in each section below. Detailed EVB simulation procedures are described in our previous work [18–20] and S2 Text. Our system consists of 10,032 atoms including 177 molaris-generated water molecules. We have done five rounds of relaxation, each lasting for 50 ns, to fully relax the system to reach the equilibrium state, the RMSD of the final round relaxation is given as S5 Fig. The final configurations were used for the subsequent EVB simulations. Free energy perturbation and umbrella sampling [21] have been used in the EVB free energy calculation by 55 frames of 20 ps each, where the time step is 1 fs. All the EVB calculations were done using the Enzymix module within the Molaris-XG package [16,22]. The detailed parameters are given in S2–S4 Tables.

### The Water Flooding (WF) Approach

The determination of internal water molecules in enzymes, especially those at the active site with charges in protein interior, is a major challenge for simulation studies [23]. The water flooding approach proved to be an efficient way to determine the most realistic configurations of water molecules by insertion of excess number of internal waters at first to over-saturate the protein, and then use the postprocessing Monte Carlo (MC) strategy to keep only the most likely configurations of the internal water molecules [24]. Development, validation and application of the water flooding approach have been described extensively [18,24,25]. Here we briefly list the key parameters we used in this study: In our simulation, the SCAAS surface constraints and the local reaction field (LRF) long-range treatment as well as polarizable ENZYMIX force field were used, and 10,000 steps of minimization followed by 200ps MD relaxation were done on our initial structure with a time step of 1.0 fs. The final structures were then used for WF simulations. During the WF simulations, a spherical hard wall was placed to prevent the exchange of the inside water molecules with the outside water molecules. The radius of the spherical hard wall for the cavity was set at 6.0 Å.

### Binding free energy calculations

The binding free energies of the substrate (CH_2_-THF) and cofactor (NAD^+^) in the presence of one magnesium ion (*Mg*_*A*_^2+^*/Mg*_*B*_^2+^) and two magnesium ions (both *Mg*_*A*_^2+^ and *Mg*_*B*_^2+^) were calculated by the semi-macroscopic version of the protein dipole Langevin dipole (PDLD) with the linear response approximation (PDLD-S/LRA). At first, we generated the MTHFD2 complex configurations with the charged and uncharged forms of solute by carrying out explicit all-atom MD simulations with the surface-constrained all-atom solvent (SCAAS) [16] and treating the long-range interaction with the local reaction field (LRF) [26]. Then we performed the PDLD/S calculations on the generated configurations. The average value was used as the consistent estimation of the binding free energy. In total, we have generated 4 configurations for the charged and uncharged states, respectively. 2 ps runs were done for each of these simulations at 300K. The dielectric constant is set to 4 for neutral protein, and the effective dielectric constant is set to 60 for the charge-charge interaction. The justification of our treatment concept has been discussed in detail [27]. This method has been established over decades to provide a reliable estimation of the binding free energies [28]. Furthermore, the binding energy between (a) $Mg_{A}^{2\text{+}}$ and Arg233; (b) $Mg_{A}^{2\text{+}}$ and the guanidino group of Arg233; and (c) $Mg_{A}^{2\text{+}}$ and the -NH_2_ of the guanidino group that directly coordinates the $Mg_{A}^{2\text{+}}$ were calculated using “prot_prot_bind” inside the level “Simplified_Electro” of the MOLARIS-XG package.

## Results

### The tale of two Mg^2+^ cations

**Fig 3:**
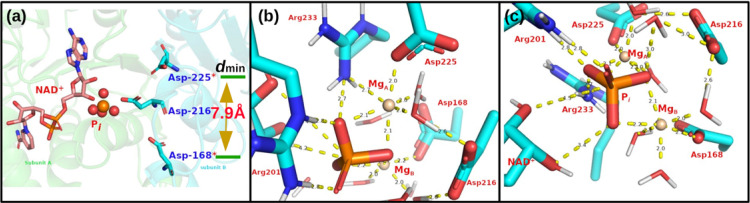
(a) NAD^+^ and P_i_ binding site from the X-ray structure (6KG2.pdb). Each monomer is labeled as subunit A (green) and B (cyan), respectively. Asp168, Asp216 and Asp225 from subunit B are colored as cyan for C atoms and shown as sticks. The minimum distance between Asp168 and Asp225 is 7.9Å. (b) The Mg_A_ binding pocket. (c) The Mg_B_ binding pocket.

The only existing site-directed mutagenesis study for MTHFD2 was done in 2005 before publication of the human MTHFD2 homodimer structures [8]. The residues that were targeted for mutagenesis experiments were Asp168 and Asp225. Asp216 is sandwiched between these two residues (see Fig 3A) but was not investigated [8]. Both D168N and D225N affect Mg^2+^ binding directly [8] and the minimum distance between Asp168 and Asp225 is 7.9 Å, which is beyond the distance required to coordinate Mg^2+^. Furthermore, we observed from preliminary simulations for one Mg^2+^ ion that the P_i_ is drifting away (see S12 Fig), suggesting the need for a second Mg^2+^ ion. Therefore, we hypothesize that there are two Mg^2+^ present to coordinate the phosphate ion. Because of this, we modeled the system with two magnesium ions and relaxed it for 50ns with the detailed stable binding pocket for $Mg_{A}^{2\text{+}}$ and $Mg_{B}^{2\text{+}}$ shown in Fig 3B and 3C. We found that Arg233, Asp225 and two water molecules coordinate $Mg_{A}^{2\text{+}}$, while $Mg_{A}^{2\text{+}}$ together with Arg233 stabilizes the two oxygens of P_*i*_. Asp168 and three water molecules coordinate $Mg_{B}^{2\text{+}}$, and $Mg_{B}^{2\text{+}}$ stabilizes P_*i*_ by forming hydrogen bonds with two oxygens of P_i_, while Arg201 coordinates directly the P_*i*_. Furthermore, Asp216 contributes indirectly to positioning and stabilization of $Mg_{A}^{2\text{+}}$, $Mg_{B}^{2\text{+}}$ and P_*i*_, through three water-mediated interactions. Our data indicates that two Mg^2+^ ions are necessary for optimal organization of the active site of MTHFD2. In addition, the binding energy between (a) $Mg_{A}^{2\text{+}}$ and Arg233; (b) $Mg_{A}^{2\text{+}}$ and the guanidino group of Arg233; and (c) $Mg_{A}^{2\text{+}}$ and the -NH_2_ of the guanidino group that directly coordinates the $Mg_{A}^{2\text{+}}$ are 7.55k_cal_/mol, 2.30k_cal_/mol, and -3.73k_cal_/mol, respectively, indicating that -NH_2_ group electrostatically contributes to the favorable interactions between Arg233 and $Mg_{A}^{2\text{+}}$.

### Enzyme catalysis

**Fig 4:**
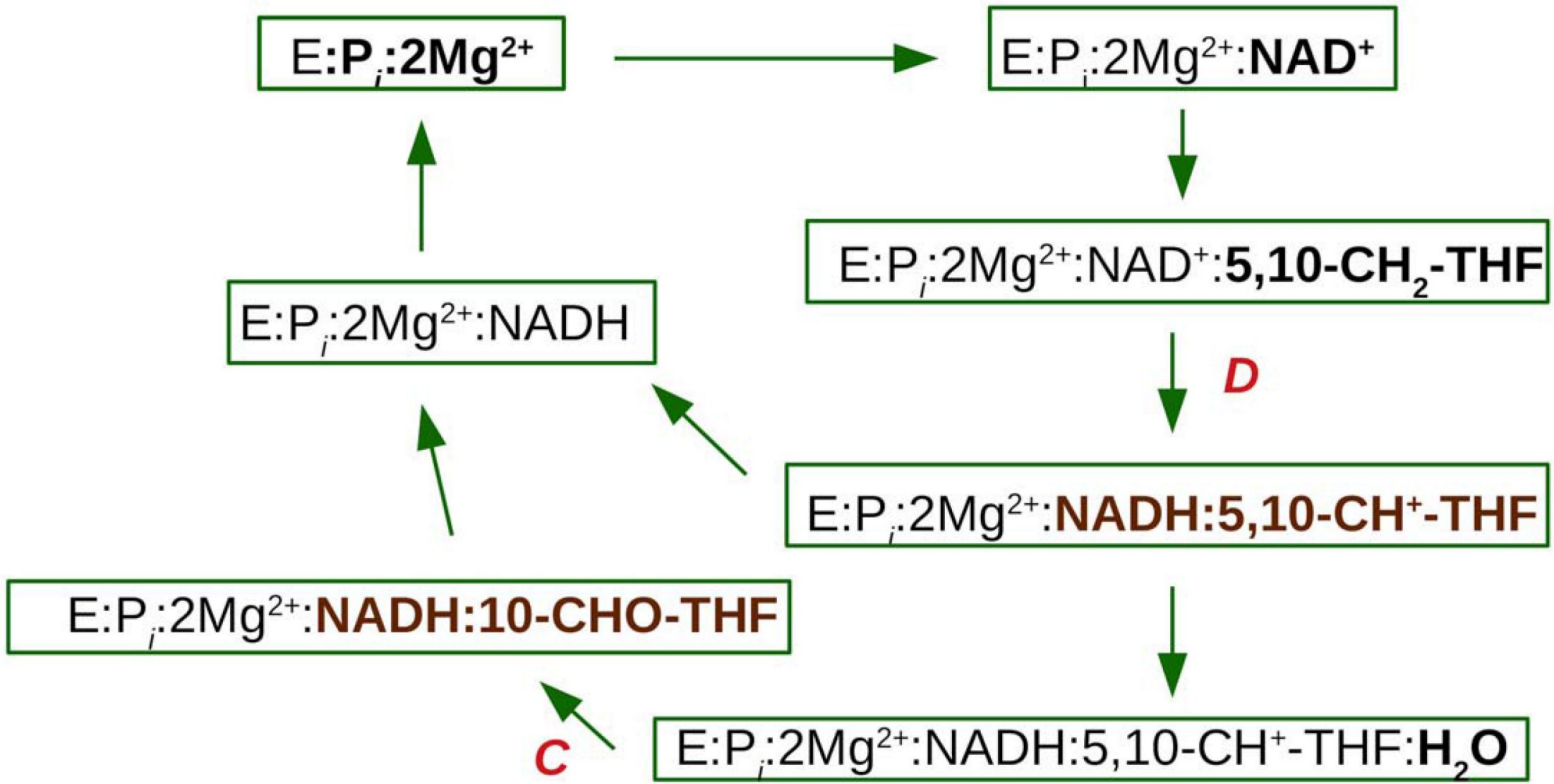
Catalytic cycle for the dehydrogenase (**D**) and cyclohydrolase (**C**) activities.

MTHFD2 combines (i) methylenetetrahydrofolate dehydrogenase and (ii) cyclohydrolase enzymatic activities. Early studies suggested that the Mg^2+^ and P_i_ ions bind to MTHFD2 first, followed by NAD^+^ and then 5,10-CH_2_-THF [11,12]. The equilibrium ordered kinetic mechanism further indicated that 5,10-CH^+^-THF is released prior NADH, while in the NADP-dependent dehydrogenase reaction the same order of addition of substrate was suggested, but NADPH is released prior to 5,10-CH^+^-THF.^14^ The catalytic cycle of the NAD^+^-dependent dehydrogenase reaction can be summarized as six important steps: (1) the ternary complex (E:P_*i*_:2Mg^2+^); (2) the NAD^+^ quaternary complex (E:P_*i*_:2Mg^2+^:NAD^+^); (3) the Michaelis complex (E:P_*i*_:2Mg^2+^:NAD^+^:5,10-CH_2_-THF); (4) the quinary product complex (E:P_*i*_:2Mg^2+^:NADH:5,10-CH^+^-THF); (5) the NADH quaternary complex (E:P_*i*_:2Mg^2+^:NADH); and (6) the release of P_*i*_, Mg^2+^ and NADH. Note that both ***D*** and ***C*** activities share the same active site and the substrate can be channeled from ***D*** to ***C*** activity. Thus, it is unnecessary to release 5,10-CH^+^-THF since it is required for the subsequent cyclohydrolase reaction. The integrated catalytic cycle including the cyclohydrolase activity is summarized in Fig 4.

#### The dehydrogenase activity

**Fig 5:**
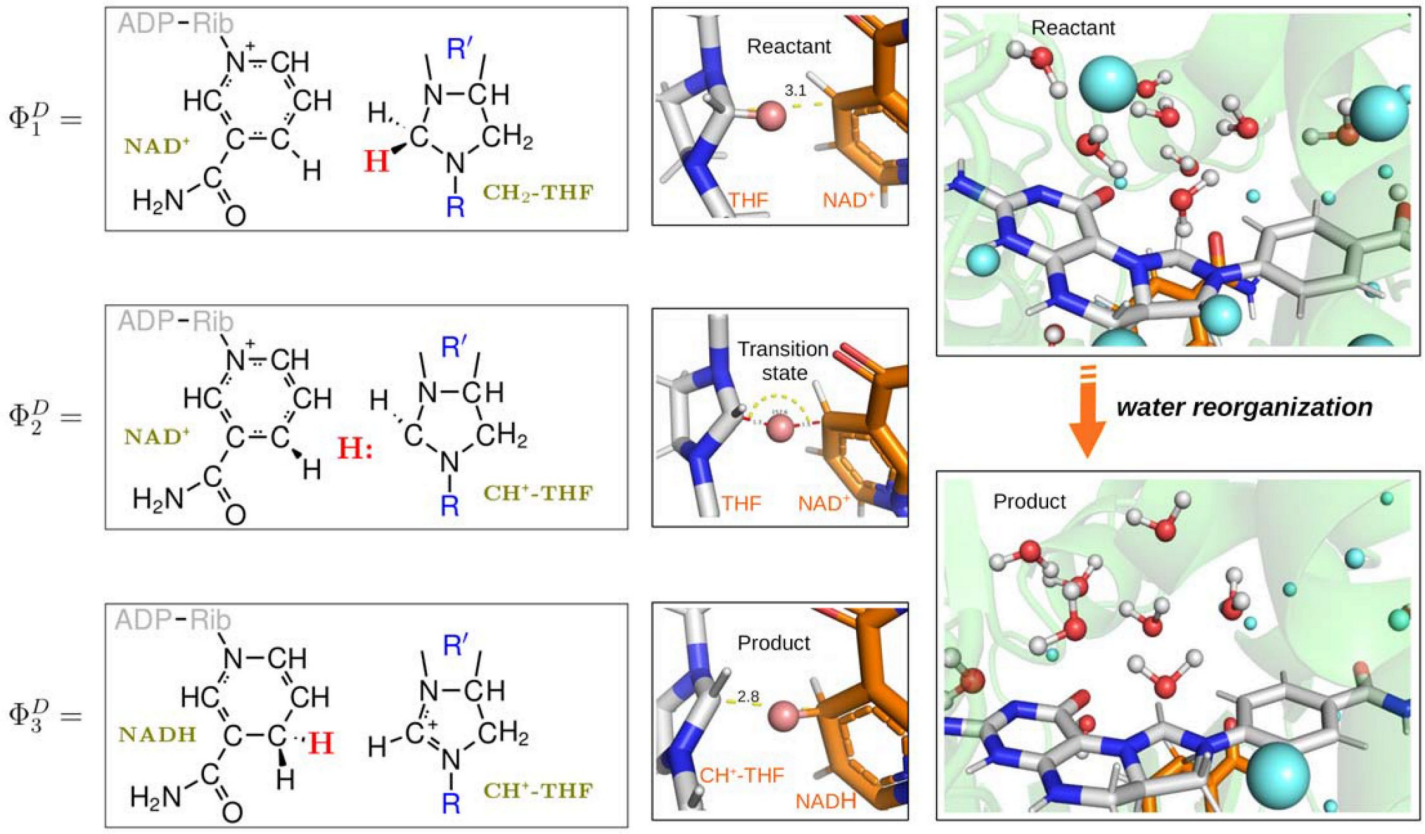
The left column shows the resonance states (Φ_1_^D^, Φ_2_^D^, and Φ_3_^D^) used to describe the dehydrogenase activity. The middle column shows snapshots of the reactant, intermediate and product state, which are depicted as stick and colored by elements (C atoms: white for THF and orange for NAD^+^/NADH). The hydroxide is shown as a sphere and colored in salmon red. In the optimized reactant geometry, the distance between the reactive C atom of CH_2_-THF and the reactive C atom of NAD^+^ is 3.1 Å, with the transferring hydrogen at 2.1 Å from the acceptor C atom. The last column shows snapshots representing the water reorganization from the reactant state to product state. The water molecules within 7Å from the reactive C atom of THF are shown as both stick and sphere and colored red for oxygen atoms, while for the water molecules beyond 7Å are only shown as blue spheres to distinguish them.

Prior to the cyclohydrolase activity, MTHFD2 catalyze the first step: the dehydrogenation of 5,10-CH_2_-THF. The computational study of the dehydrogenase catalytic mechanism of MTHFD2 is described by three EVB resonance states (Φ_1_^D^, Φ_2_^D^, and Φ_3_^D^) that represent the reactant, intermediate, and product states (Fig 5). The hydride (H:) transfers from 5,10-CH_2_-THF to NAD^+^ in a *proton-coupled electron transfer* (PCET) way. This is electronically nonadiabatic with weak coupling between the reactant and product electronic states and ends with the electron delocalized in the pyridine ring of the nicotinamide moiety of the NADH. The reactive *C* atoms of THF and NAD^+^ in the optimized reactant geometry are at a 3.1Å distance from each other, with the transferring hydrogen at 2.1Å from the acceptor *C* atom. The π-π stacking interaction between the NAD^+^ pyridine ring of nicotinamide moiety and THF’s imidazoline ring connects the two reactants in the active site. The π-π stacking between Tyr84 and THF’s *p*ABA moiety orientating the THF binding and the flexibility of the (poly)glutamate tail of THF, was observed during simulations and can promote the formation of the transition state. In addition, PCET not merely involves substantial protein charge redistribution (from THF to NAD^+^) but also involves ordered solvent reorganization which is important for the subsequent reaction to proceed (illustrated in Figs 5 and S9).

We have calculated the binding energy of THF, NAD^+^, and PO_4_^3-^ in the presence of one magnesium (either $Mg_{A}^{2\text{+}}$ or $Mg_{B}^{2\text{+}}$), two magnesium, and no magnesium, respectively (Table 1). We found that the presence of magnesium does not affect THF binding but there was a moderate effect on NAD^+^ binding and a strong effect on PO_4_^3-^ binding. Our conclusion is that the presence of Mg^2+^ is important for PO_4_^3-^ binding. We have observed an interesting result from our free energy calculations concerning the reaction kinetics. Since we have no experimental information of the reactions in solution, we have calibrated our EVB results with the reference water run using the B3LYP function with cc-pVTZ basis sets. The presence of one magnesium ion ($Mg_{B}^{2\text{+}}$) seems more energy favorable than the two magnesium system, which is an important insight because the two magnesium ions may contribute significantly to PO_4_^3-^ binding while slowing down the reaction kinetics.

**Table 1:** Calculated binding energy and free energy (k_cal_/mol) for the dehydrogenase activity of MTHFD2. The $\Delta G_{obs}^{D,‡}$ is calculated based on the experimental value k_cat_ = 12.4 ± 0.71s^-1^ using transition-state theory.^10^

Systems	ΔG^*D*,‡^	ΔG^*D*^	$E_{binding}^{THF}$	$E_{binding}^{NAD^{\text{+}}}$	$E_{binding}^{PO_{4}^{3-}}$
MTHFD2 with 2 Mg^2+^	24.1	4.7	-0.50	-10.45	-97.56
MTHFD2 with $Mg_{A}^{2\text{+}}$	22.5	13.4	-0.74	-0.22	-37.66
MTHFD2 with $Mg_{B}^{2\text{+}}$	16.9	1.9	-0.34	-5.72	-42.67
MTHFD2 without Mg^2+^	29.3	3.8	-0.82	5.37	-8.53
$\Delta G_{obs}^{D,‡}=16.0∼16.1$					

#### The cyclohydrolase activity

Both MTHFD2 and MTHFD2L exhibit robust cyclohydrolase activity [9,10], which are independent of their dehydrogenase activity and NAD^+^ does not affect the reaction. Due to the relatively fast rate of the reaction and high absorbance of the substrate, it is hard to determine accurate *k*_*cat*_ and *K*_*M*_ values experimentally [9].

The cyclohydrolase activity involves the nucleophilic addition of the hydroxide anion to the imidazoline moiety and the opening of the hydrated imidazoline ring. No mechanism has been proposed for this reaction, indicating a void of information that needs to be filled.

**Fig 6:**
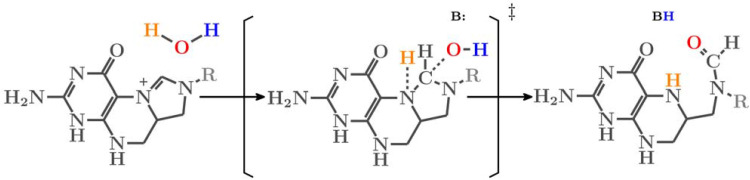
Proposed cyclohydrolase reaction mechanism. ^‡^ represents transition state.

For catalysis, the opening of the imidazoline ring has three requirements: (i) activation of a water molecule for nucleophilic addition; (ii) the formation of a putative hydrated imidazoline intermediate; and (iii) a base (B:) to promote the scission of the central bond by abstraction of the hydrogen from the putative hydrated imidazoline intermediate. Even though the nature of the protein active site cavity is an aprotic environment, the Lys88 side chain provides polar interactions that anchors the oxygen of the pterin group from 5,10-CH^+^-THF by direct hydrogen bonding, and further interacts with the side chain of Gln132. However, neither Lys88 nor Gln132 can be the base to promote the scission of the central bond by withdrawing the hydrogen from the intermediate (S8 Fig). Here, we used the water flooding approach to estimate the number of water molecules that can be present at the active site. We found an average of 8 water molecules located right at the active site, which is significant enough to form a protic proton-recycle system (see Figs 5 and S9) that could facilitate a low-barrier hydrogen transfer/exchange. The possibility of forming a proton-relay system that recycles/exchanges the hydrogen to favor the cyclohydrolase reactions is tantalizing because it resolves which base abstracts the hydrogen from the hydrated imidazoline intermediate. Using the hydrated active site and computational approaches, we observed a concerted reaction mechanism for the hydration of the imidazoline ring (Fig 6). One hydrogen from water was abstracted by the pyridine-nitrogen atom of the imidazoline ring, while the hydroxide of the water molecule was attracted to the pyrrole-nitrogen atom. Furthermore, it is relatively inexpensive to transport water molecules from bulk water to any site in the protein. However, transport of OH^-^ is probably more demanding in terms of free energy. The rate limiting step of the cyclohydrolase reaction may be the formation of the hydroxide ion that attacks the imidazoline ring when the tetrahedral transition state is formed. The activation free energy is then the sum of the free energies for both processes. Formation of the hydroxide ion is heavily pH dependent. At room temperature and pH value of 7.4 the estimated energy required to form the hydroxide is 11.3 k_cal_/mol [based on K_B_Tln(10)*(15.7−pH)] [29], indicating that this reaction is likely to happen. Since the pH inside the mitochondria is slightly above 7 (range from 7.0 to 7.8) [30], this alkaline environment may facilitate the abstraction of the hydrogen from hydroxide to facilitate the scission of the central bond.

Furthermore, it would not be catalytically “wise” to release the 5,10-CH^+^-THF immediately after the dehydrogenase reaction is completed since it needs to be at the same active site for the subsequent cyclohydrolase reaction given that a single site is used for both dehydrogenase and cyclohydrolase activities [11]. Hence, we suggest that 5,10-CH^+^-THF is channeled from the previous dehydrogenase reaction to the subsequent cyclohydrolase reaction, which is also supported by a study of its cytosolic counterpart MTHFD1 [31]. A conformational change, particularly the solute reorganization during the dehydrogenase activity, may be responsible for the activation of the cyclohydrolase process. Such a mechanism would increase the catalytic efficiency of MTHFD2.

### To be or not to be NAD^+^-dependent

**Fig 7:**
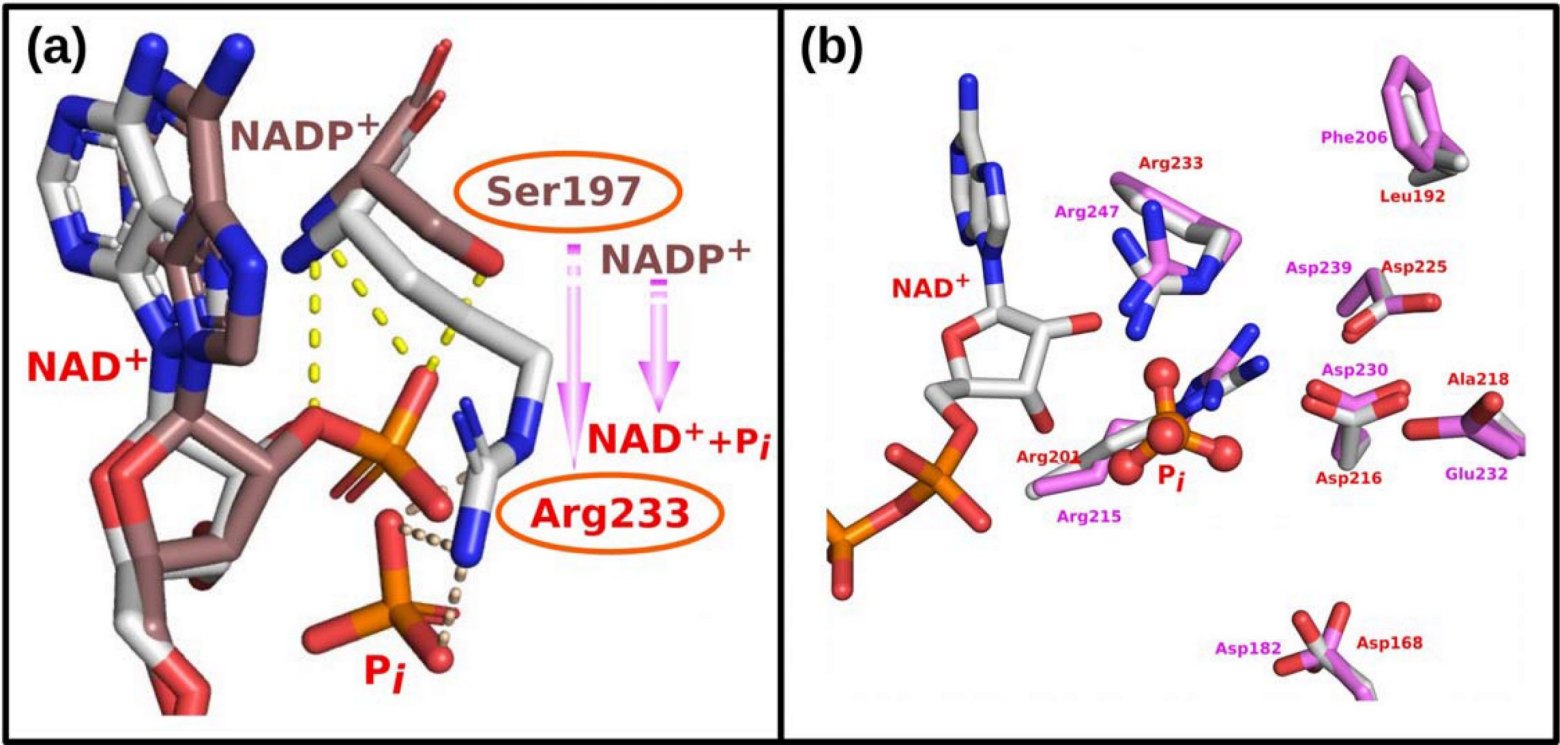
(a) Superimposition of the NAD(P)^+^ binding pockets with regards to the dehydrogenase activity. The C atoms of MTHFD1 (PDB: 6ECQ) are colored in brown, and those of MTHFD2 (PDB: 6KG2) are colored in white. Switching from Ser197 to Arg233 defines a key force in binding 2′-phosphate of NADP^+^ rather than phosphate ion (P_i_). (b) Superimposition of the MTHFD2 and MTHFD2L NAD^+^ binding pockets. Key residues involved in binding are shown as sticks and colored red for MTHFD2 and pink for MTHFD2L.

The cofactor preference of MTHFD2 for NAD^+^ (rather than NADP^+^) in the dehydrogenase activity is believed to promote “a more thermodynamically favorable pathway to balance the pools of 10-formyl-THF during development” [32]. Sequence and structure comparison of MTHFD1 and MTHFD2 (Fig 7) indicated that Ser197 is a key factor in binding the 2′-phosphate of NADP^+^, while Arg233 is important to bind the phosphate ion. The change from Ser197 in MTHFD1 to Arg233 in MTHFD2, may enable switching the dehydrogenase activity from NADP^+^-dependent to NAD^+^-dependent. Mutagenesis of MTHFD2 indicates that the Arg233Ser mutant almost completely lost the NAD^+^-dependent dehydrogenase specific activity with a 50% decrease in NADP^+^-dependent dehydrogenase specific activity [8]. Furthermore, since MTHFD2L has been reported to use either NAD^+^ or NADP^+^ as cofactor, we have used homology modeling to build the MTHFD2L structure and superimposed it with MTHFD2 (for sequence alignment see S7 Fig). Residues near the NAD^+^ binding pocket are well-conserved, while more distant residues vary, which contributes to the difference in catalytic efficiency between the MTHFD2 and MTHFD2L. Since earlier kinetic studies have shown that the ions bind MTHFD2 before NAD^+^ [11,14], the binding of ions also selectively prevents NADP^+^ binding due to the steric occupation of the 2′-phosphate group of NADP^+^ by ions.

### Implication for drug discovery

As a good target for anticancer drug development, we need a drug specifically targeting MTHFD2 [5], without affecting its cytosolic homolog MTHFD1 and the mitochondrial homolog MTHFD2L. Because MTHFD1 is expressed in normal tissues and most abundantly in the liver [33], inhibition of MTHFD1 would cause most likely unwanted side effects. MTHFD2L, sharing 60 ~ 65% identity with MTHFD2 and with a highly conserved substrate and cofactor binding pocket, is expressed in all tissues examined, with the highest expression levels in brain and lung in both humans and rodents [9,34]. Therefore, a selective inhibitor for MTHFD2 over MTHFD1 and MTHFD2L is desirable as a lead for drug discovery to warrant successful clinical investigations. There are two apparent pockets in MTHFD2 for structure-based drug discovery: one is the cofactor (NAD^+^) pocket and the other is the substrate (CH_2_-THF) pocket. Since we know that NAD(P)^+^-dependent enzymes are ubiquitously expressed in metabolism and other cellular processes [35], inhibition of NAD(P)^+^ may induce significant toxicity. In addition, the substrate pocket is also present in other enzymes of the THF-mediated one-carbon metabolic pathway such as SHMT1/2, MTHFD1 and MTHFD2L. Therefore, interventions in the THF-mediated pathway in normal cells could induce undesired side effects. Since these two pockets are not suitable for specific inhibitors, we searched for novel pockets for drug binding. These studies uncovered a novel integrated pocket, different from the current cofactor and substrate clefts, which is formed at the interface between two monomers during dimerization (illustrated by balls in S4 Fig). By exploiting this structurally less conserved allosteric site of MTHFD2, we would be able to develop new inhibitors to help cancer patients. A recent effort has been made into this direction with Xanthine derivatives binding at the allosteric binding site of MTHFD2, which prevents the binding of the cofactor and phosphate [36]. In order to identify compounds that could potentially bind to the novel dimerization pocket, we screened compounds from the ZINC15 database. Our preliminary screening identified 3 clusters of compounds (see S1 Table) that have potential to bind this structurally less conserved allosteric site. These compounds neither fully occupy the substrate- nor the cofactor binding site, but may protrude to the above two canonical binding sites which prevents the binding of either substrate or cofactor. Alternatively, these compounds may bind at the same time with the substrate and cofactor. Further structural and in vivo studies are required to validate these proposed compounds to elucidate the mechanism of inhibition and further develop them into candidate drugs.

## Discussion

Even though it has been well recognized that there is an absolute requirement for Mg^2+^ in the MTHFD2 dehydrogenase activity, it has been a challenge to determine the location of the Mg^2+^ by X-ray crystallography due to the extremely low electron density and the small size of Mg^2+^ [37]. Scrutiny of the crystal structure of MTHFD2 indicates that the closest distance between Asp168 and Asp225 is 7.9Å (Fig 3A), and another residue, Asp216, is sandwiched between Asp168 and Asp225 (PDB ID: 6KG2), which leads us to propose a model of two Mg^2+^ assisting in P_i_ binding. This model is further supported by the fact that one Mg^2+^ alone is not capable of stabilizing the P_*i*_ in our detailed simulation studies. Our work has captured the presence of two Mg^2+^ cations and provided a clear picture of how they coordinate and stabilize the inorganic phosphate as well as how they contribute to catalysis. Furthermore, Asp168, Asp216, and Asp225 from monomer A, and Arg201 and Arg233 from monomer B, collectively coordinate the two Mg^2+^ and P_i_, which explains why MTHFD2 functions as a homodimer and homodimerization occurs by antiparallel interaction of the two NAD^+^-binding domains.

The dehydrogenase activity transfers two electrons coupled to the proton (H:) and this involves both electron relocalization featuring substantial protein charge redistribution and ordered solvent reorganization which activates the subsequent cyclohydrolase reaction. The cyclohydrolase activity features the opening of the nucleophilic attacked imidazoline ring, which is facilitated by the proton-recycling-network present at an aprotic pocket enabling low-barrier proton exchange. Furthermore, we were able to show that the Ser233 to Arg switch determines the cofactor preference from NAD^+^ to NADP^+^.

MTHFD2 is an important drug target lacking inhibitors in clinical trials or with FDA-approval. Our detailed structural analysis and mechanisms studies have uncovered (a) the presence and location of two Mg^2+^ ions; (b) the dehydrogenase activity orchestrated by a proton-coupled electron transfer not only facilitating the proton transfer but also promoting the subsequent cyclohydrolase reaction; (c) a putative cyclohydrolase reaction mechanism; and (d) the key factors that determine the cofactor preference. Our study supports the importance of Mg^2+^ in the enzymatic activity of MTHFD2. In order to better understand the roles of two Mg^2+^ ions system, we dissected it into two interconnected roles: one is the structural role featuring the binding energy of substrate and cofactors and found it significantly contributes to the stabilization of the cofactor PO_4_^3-^_._ The other one is the functional role, where our study indicates the disadvantage of two magnesium ions because it slightly slows down the reaction kinetics. Our detailed structural information has located the position of the two magnesium ions in MTHFD2, which provides a framework for the future characterization of the roles, the order of binding, and mode of binding of these two Mg^2+^ ions, as well as the underlying mechanism. We also systematically reviewed the experimental evidence of the two Mg^2+^ system and its implications for catalysis and given in S3 Text. In addition, the reaction mechanism has been investigated in an integrated way, rather than as a combination of isolated reaction steps, which may shed new light on the drug target MTHFD2.

In our study, we focused on the reaction mechanism of MTHFD2. However, a few more discussions about the computational biochemical approaches we used could be of interest. As we described earlier, the initial structures we used are based on homology modeling with known templates. The active site was reconstructed using local QM/MM optimizations. One well-known challenge of QM/MM is the convergence, which is a limitation of our study. The active site we reconstructed may not fully represent the active site in the active state. The extensive studies done by Ryde and co-workers found that convergence is pertinent to the size of QM region [38]. Extensive studies with different sizes of QM region may help find the true active sites. Himo and Siegbahn have proposed the quantum chemical cluster approach which significantly improve the convergence issue on solvation energy [39]. In our work, we mainly used EVB approaches [21,32,33]. This approach features a classical force field within a valence bond based quantum-mechanical framework, and provides converged results. It has been widely used in interpreting the reaction mechanism [18–20,40–45], exploring inhibition [46], and redesigning enzymes with enhanced activities [47].

The nature of the hydride transfer has attracted a plethora of studies including the study of the temperature dependence of the kinetic isotope effects (KIEs) [47, 48]. One reason to study the KIEs of the hydride transfer is that the hydride is heavier compared with the electron and proton transfer. The heavier isotope is associated with lower vibrational frequency and more energy is required to reach the transition state, thus the reaction rate is slower. Kinetic studies of mutant methylenetetrahydrofolate dehydrogenase/cyclohydrolase activities of MTHFD1 have indicated that the change of kinetic isotope ratio in the dehydrogenase activity, and that Tyr52 (corresponding to Tyr84 in MTHFD2) plays a role in binding and orientation of the substrate. Furthermore, Lys56 (corresponds to Lys88 in MTHFD2) was proposed to possess a catalytic role [49, 50]. However, after analyzing the data (Table 4 in Ref. [50]), we think during the dehydrogenase activity, Lys56 contributes more to the binding (the K_M_ of K56E mutant is more than 6 times higher than wild type) and it does not have noticeable catalytic role based on the kinetic isotope ratio which is 2.96 vs. 2.87 compared to wild type. Even after careful examination of the role of Lys88 in the cyclohydrolase activity, we can not unambiguously exclude a catalytic role of Lys88. One possibility is that since we are still searching for the candidate base that abstracts the hydrogen, which is a limitation of our studies, that we need to examine the possibility that Lys88 acts as this base. In this study, we have hypothesized a proton-relay system that recycles/exchanges the hydrogen to favor the cyclohydrolase reaction by abstracting that blue H in the alkaline environment (pH = 7 ~ 7.8).

Our work not only contributes to the understanding of the binding mode and catalytic mechanism of the drug target MTHFD2, which is relevant not only for further studies on drug discovery, but also provides new cues concerning the coupling of metal and cofactor binding in the catalytic steps of other enzymes [51].

### Limitations

We provided a general description of the cyclohydrolase activity and the quantitative description of the cyclohydrolase activity will be of great interest for future studies. A quantitative description could not merely test the proposed mechanism of the cyclohydrolase reaction but also could be indicative of a non-optimal structure/composition of all players in the active site, including Mg^2+^ ions and water molecules.

We cannot unambiguously conclude there are two Mg^2+^ ions present and exclude the one Mg^2+^ ion option. Our assumption of two Mg^2+^ based on the mutagenesis studies published in 2005, and our simulation indicate that one Mg^2+^ is incapable of stabilizing the phosphate ion. Furthermore, our free energy calculation indicates a lower activation energy is achieved when only one Mg^2+^ is present, which indicates the second Mg^2+^ may contribute to the binding but not necessarily catalysis.

Normally the proton-coupled electron transfer would mean that the Born-Oppenheimer approximation will be used to separate the proton and the electron, and the electronic Schrödinger equation must be solved for various nuclear positions and forms the potential energy (hyper)surface. Since a number of degrees of approximation would be involved in this approach, the inaccuracy may increase. In our work we assumed that the proton transfer is concerted with the transfer of the electron lone pair and therefore only one Born-Oppenheimer surface needed to be considered. Of course this simplification may be a limitation and the approaches of Hammes-Schiffer and coworkers to PCET of are of great interest [52].

Finally, the computational approaches that we have used are based on calculations and hypothetical scenarios and are applied to only limited parts of the enzyme. This is mainly due to the complexity of the calculations and because they are computationally expensive. Nevertheless, many results applying similar computational approaches as used here, have been validated elsewhere [40,53] and therefore the majority of our results will shed in-depth insight about the structure and mechanism of MTHFD2.

## Supporting information

S1 Fig The superimposed snapshots of all crystallized MTHFD2 structures (1ZN4, 5TC4, 6JIB, 6JID, and 6KG2). The proteins are shown as surface. NAD^+^ and inhibitors are shown as sticks. Phosphate is shown as spheres.
(TIF)

S2 Fig Representative docked pose with 5,10-CH_2_-THF and NAD^+^ *in a* catalytic feasible conformation. MTA, 5,10-CH2-THF and Arg43 are shown as sticks. P_i_ is shown as spheres.
(TIF)

S3 Fig The chemical structure of NAD^+^ and 5,10-CH_2_-THF. (TIF)

S4 Fig (A) Cofactor binding groove (NAD^+^), substrate groove (THF), and the homodimer groove integrated with a partial substrate groove which is filled with balls for illustration purposes. (B) and (C) are our preliminary screening poses for illustration purposes.
(TIF)

S5 Fig Depiction of the atom (in region I of our EVB simulations) with ID. (TIF)

S6 Fig RMSD of the heavy atoms of last round relaxation. (TIF)

S7 Fig Sequence alignment of MTHFD2L and MTHFD2. The residues within 12*Å* from the NAD^+^ binding pocket were analyzed and colored blue (conserved between the two) and red (different between the two).
(TIF)

S8 Fig The snapshot of the protic proton-recycle-system. (TIF)

S9 Fig A snapshot to illustrate the catalytic process. The proton transfer from 5,10-CH_2_-THF to NAD^+^ is accompanied by the charge redistribution with the dynamics of the water molecules featuring the re-ordering of water molecules to facilitate the subsequent cyclohydrolase reaction.
(TIF)

S10 Fig (a) Overview of the structure of Ketol-acid reductoisomerase (PDB ID: 4KQX). (b) The distance between two Mg^2+^ ions is 4.3Å. (c) The residues that coordinate the two Mg^2+^ ions system.
(TIF)

S11 Fig Snapshot to illustrate the NAD^+^ does not interact with protein from the structures of the riboswitch (PDB ID: 7D7V, 7D7W and 7D81). The NAD^+^ is shown as sticks while the Mg^2+^ are shown as spheres.
(TIF)

S12 Fig P_i_ is drifting away when there is only one Mg^2+^. (TIF)

S1 Table The compounds that bind the structurally less conserved homodimer groove (allosteric sites) from our preliminary screening. (XLSX)

S2 Table Atom type and partial charges used in our EVB study. (XLSX)

S3 Table EVB bond parameters. (XLSX)

S4 Table Lennard Jones potential. (XLSX)

S5 Table The proteins whose crystallized structures contain Mg^2+^ and/or NAD^+^. Note that the colored ones are the ones with two Mg^2+^ ions in close proximity of NAD^+^.
(XLSX)

S1 Text Modeled systems. (DOC)

S2 Text Empirical valence bond (EVB) simulations. (DOCX)

S3 Text Experimental evidence of the two Mg^2+^ system and its implications for catalysis. (DOC)
