# Supplementary figures and images for: The catalytic mechanism of the mitochondrial methylenetetrahydrofolate dehydrogenase/cyclohydrolase (MTHFD2)
Source: PLoS Comput Biol. 2022 May 25;18(5):e1010140. doi: 10.1371/journal.pcbi.1010140 (PMC9173628; doi:10.1371/journal.pcbi.1010140)

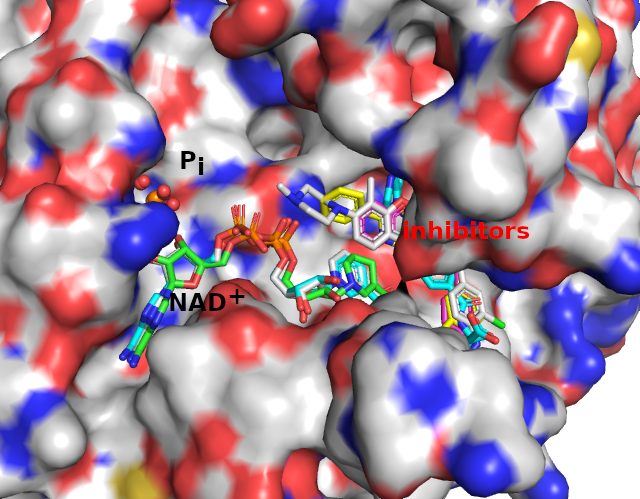

Supplement: S1 Fig — The proteins are shown as surface. NAD+ and inhibitors are shown as sticks. Phosphate is shown as spheres. (TIF) [file pcbi.1010140.s001.tif]

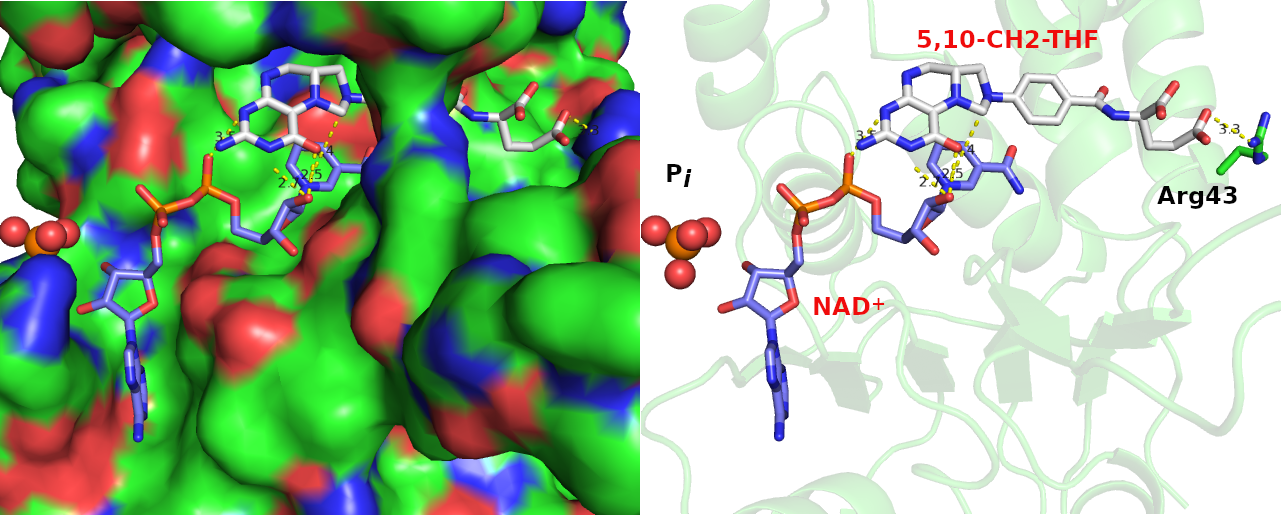

Supplement: S2 Fig — MTA, 5,10-CH2-THF and Arg43 are shown as sticks. Pi is shown as spheres. (TIF) [file pcbi.1010140.s002.tif]

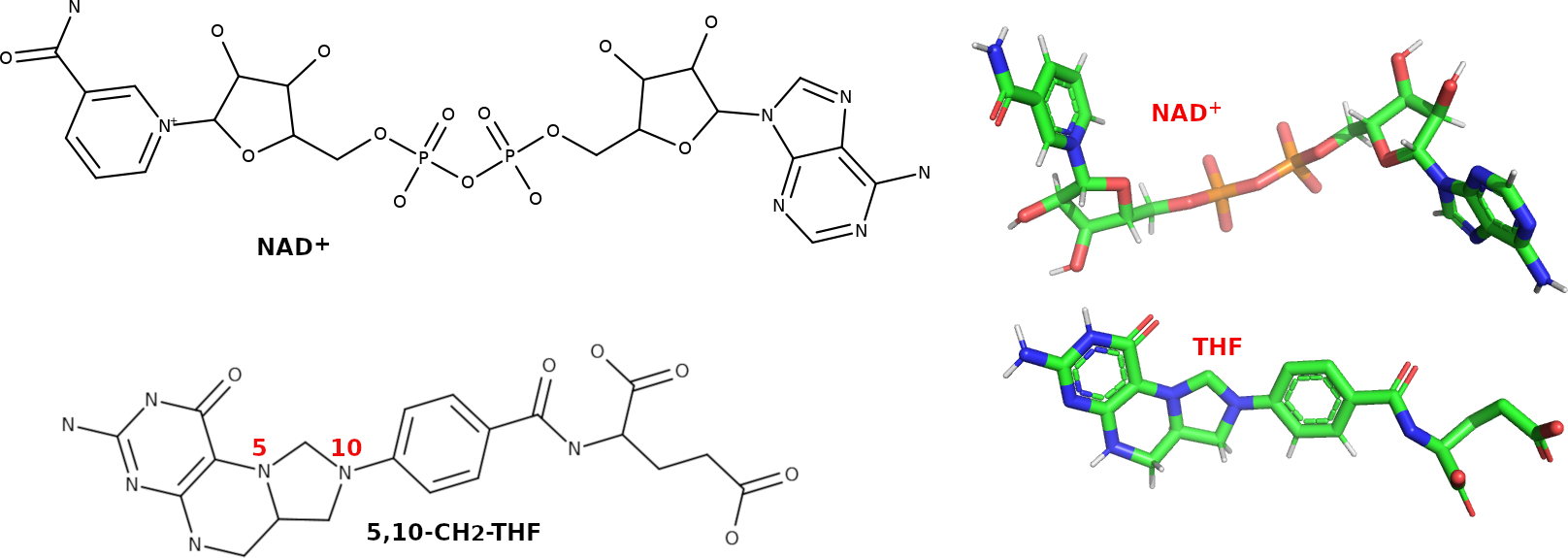

Supplement: S3 Fig — (TIF) [file pcbi.1010140.s003.tif]

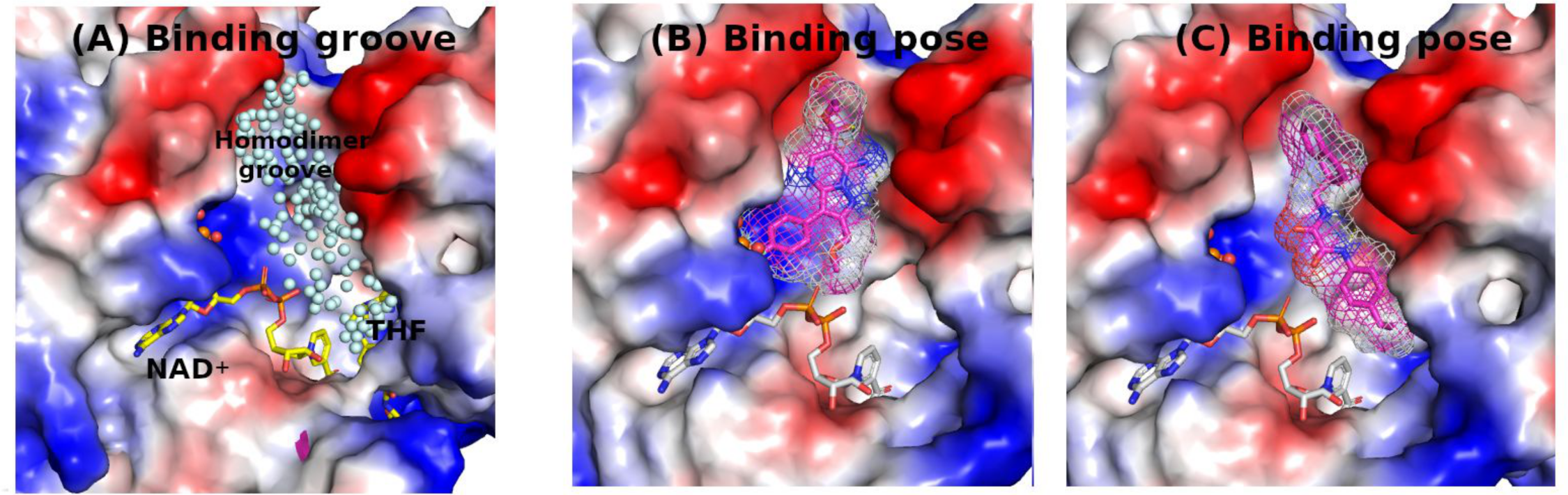

Supplement: S4 Fig — (A) Cofactor binding groove (NAD+), substrate groove (THF), and the homodimer groove integrated with a partial substrate groove which is filled with balls for illustration purposes. (B) and (C) are our preliminary screening poses for illustration purposes. (TIF) [file pcbi.1010140.s004.tif]

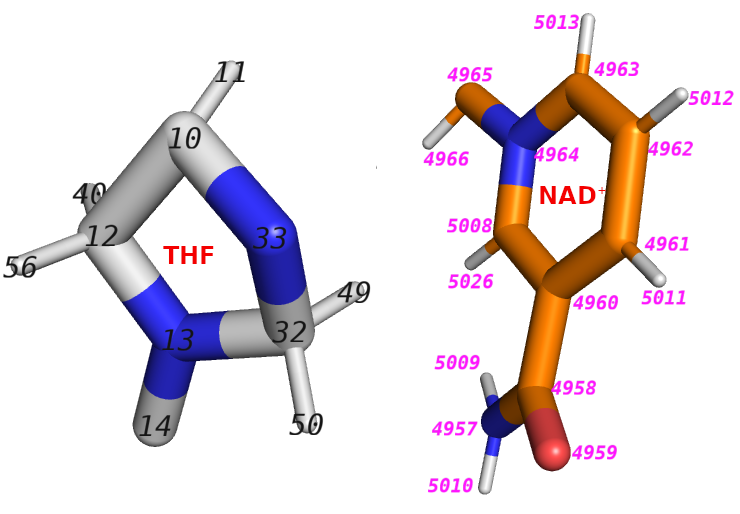

Supplement: S5 Fig — (TIF) [file pcbi.1010140.s005.tif]

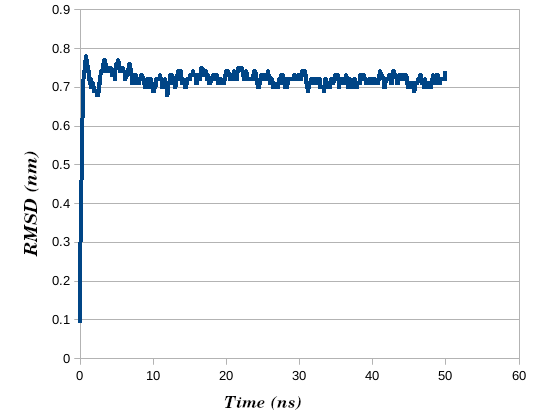

Supplement: S6 Fig — (TIF) [file pcbi.1010140.s006.tif]

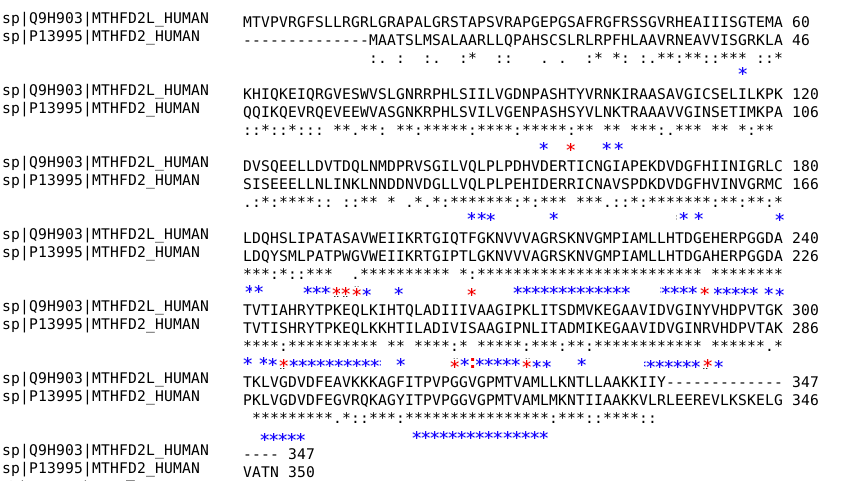

Supplement: S7 Fig — The residues within 12Å from the NAD+ binding pocket were analyzed and colored blue (conserved between the two) and red (different between the two). (TIF) [file pcbi.1010140.s007.tif]

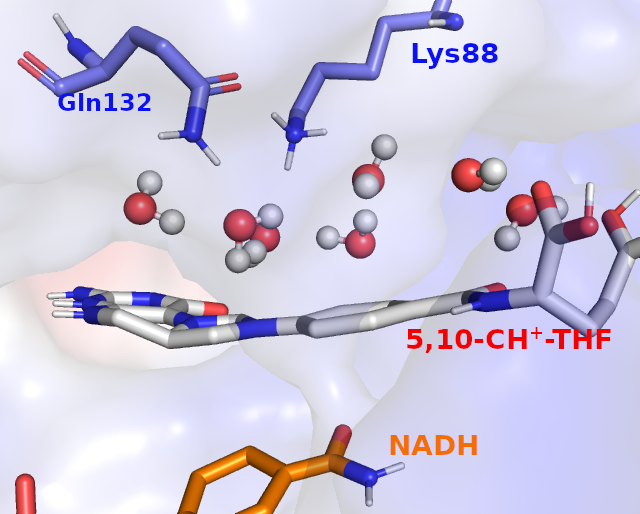

Supplement: S8 Fig — (TIF) [file pcbi.1010140.s008.tif]

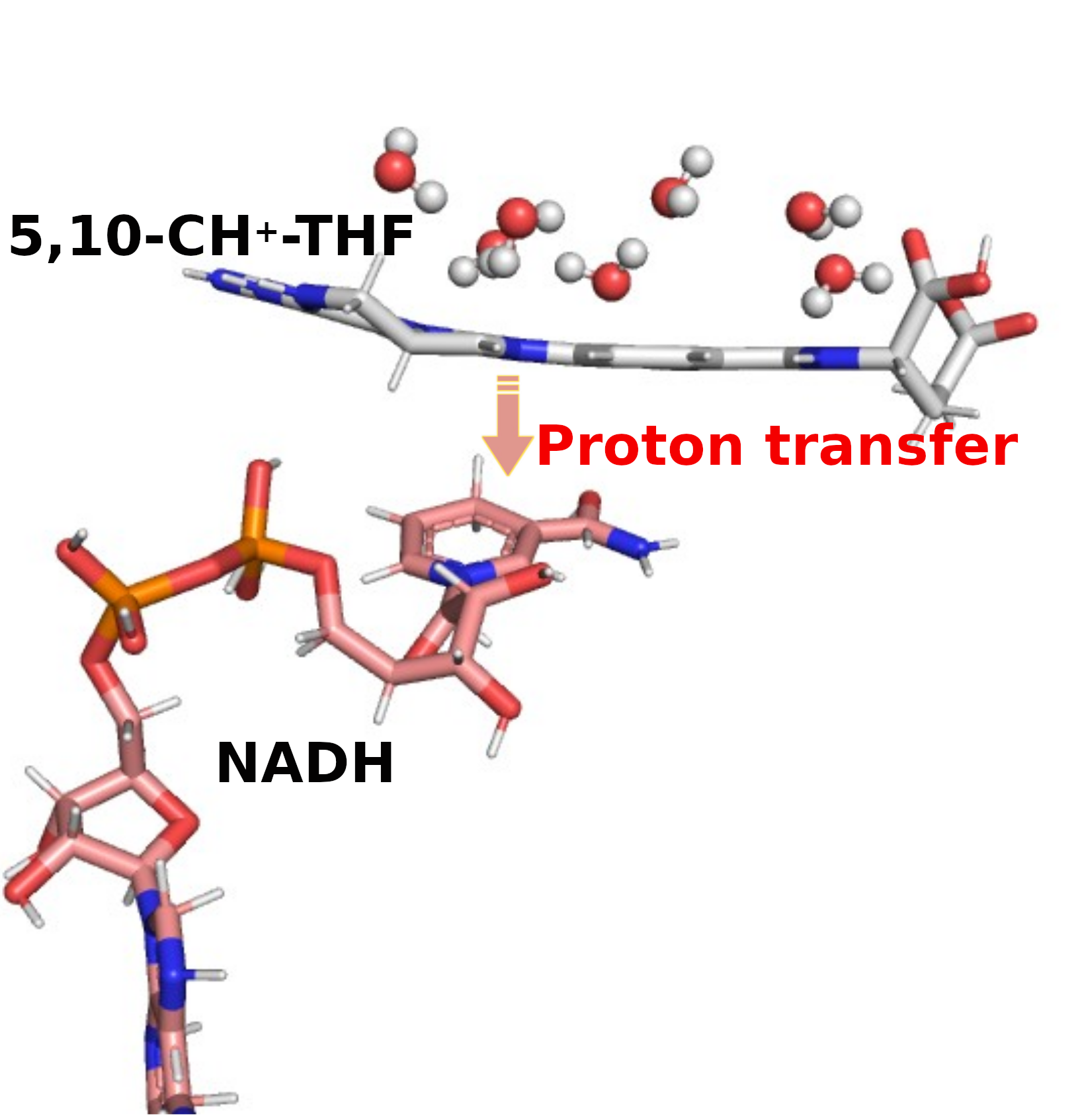

Supplement: S9 Fig — The proton transfer from 5,10-CH2-THF to NAD+ is accompanied by the charge redistribution with the dynamics of the water molecules featuring the re-ordering of water molecules to facilitate the subsequent cyclohydrolase reaction. (TIF) [file pcbi.1010140.s009.tif]

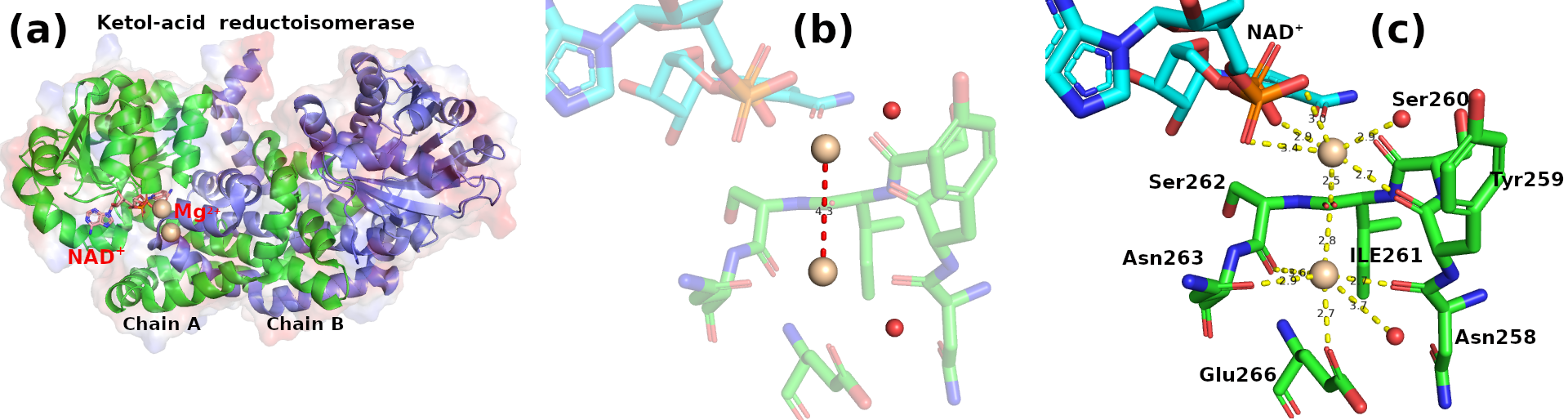

Supplement: S10 Fig — (a) Overview of the structure of Ketol-acid reductoisomerase (PDB ID: 4KQX). (b) The distance between two Mg2+ ions is 4.3Å. (c) The residues that coordinate the two Mg2+ ions system. (TIF) [file pcbi.1010140.s010.tif]

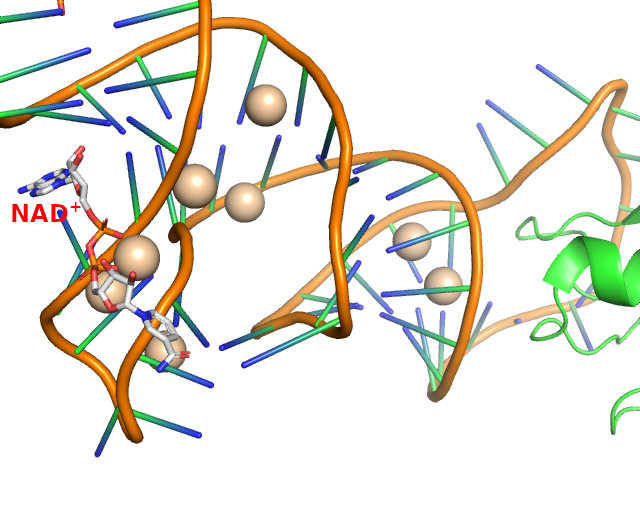

Supplement: S11 Fig — The NAD+ is shown as sticks while the Mg2+ are shown as spheres. (TIF) [file pcbi.1010140.s011.tif]

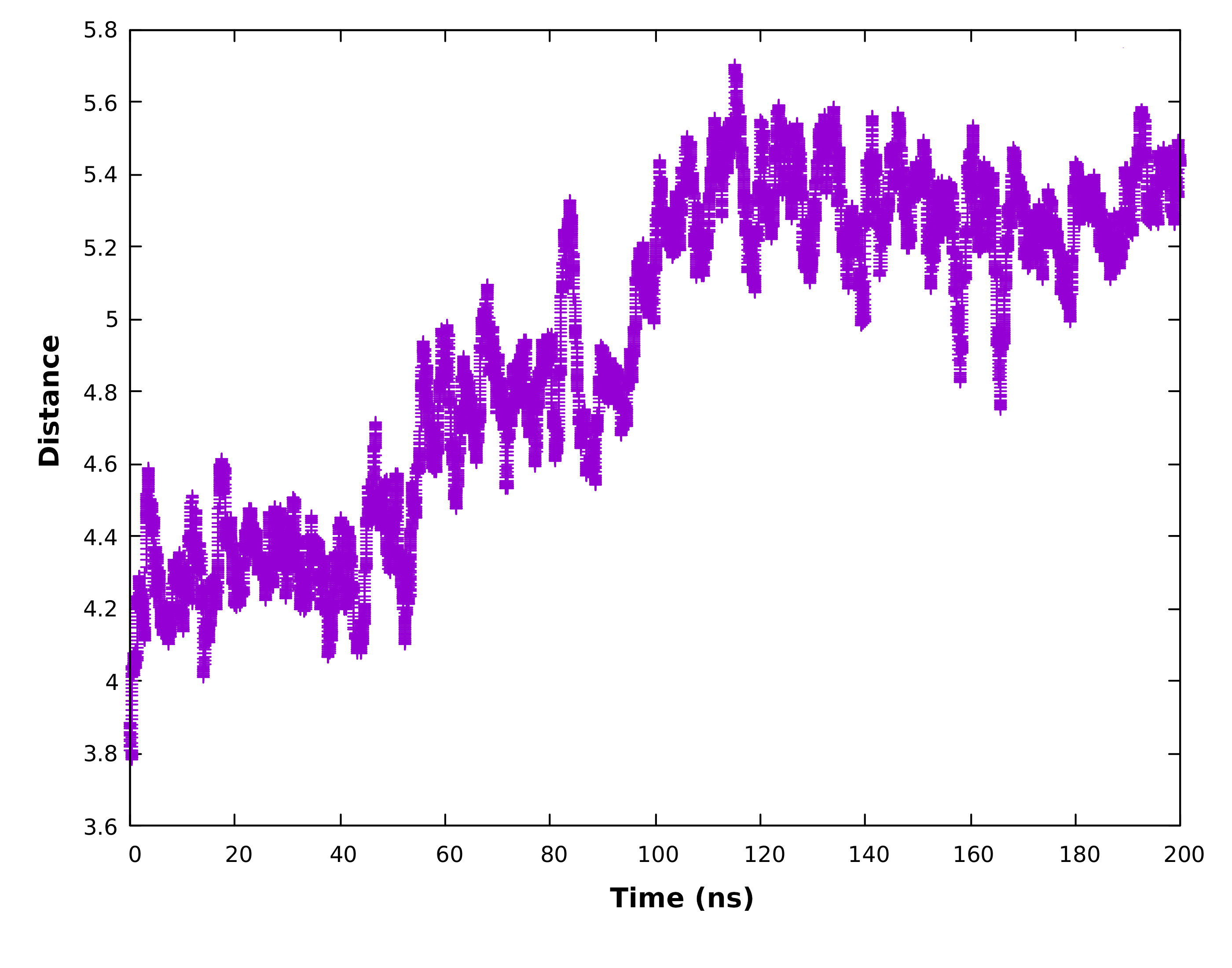

Supplement: S12 Fig — (TIF) [file pcbi.1010140.s012.tif]
