# Supplementary material for: The catalytic mechanism of the mitochondrial methylenetetrahydrofolate dehydrogenase/cyclohydrolase (MTHFD2)
Source: PLoS Comput Biol. 2022 May 25;18(5):e1010140. doi: 10.1371/journal.pcbi.1010140 (PMC9173628; doi:10.1371/journal.pcbi.1010140)
Supplement: S1 Text — (DOC) [file pcbi.1010140.s018.doc]

### **M**odeled systems

*Structure background*

In 2016, the first crystallization of MTHFD2 in complex with NAD^+^, inorganic phosphate (P_i_), and LY345899 was solved at 1.89Å and provides a reliable template (5TC4.pdb) to start structure based inhibitor studies [1]. The latest structure of MTHFD2 in complex with DS44960156 (6JIB.pdb), Compound 1 (6JID.pdb), and DS18561882 (6KG2.pdb) as well as cofactors (NAD^+^ and P_i_) were solved at 2.25Å and bears similarities with the first identified structure. With all these structures, the exact location of Mg^2+^ and its molecular interactions are still not known. Since MTHFD2 functions as dimer, we have reconstituted the active site of the MTHFD2 in a homodimeric complex (MTHFD2·NAD·P_i_·Mg^2+^·P_i_·THF) with inorganic phosphate, NAD^+^, and substrate using homology modeling.

*Construction of the two magnesium system*

In order to investigate the crystallized structure of NAD^+^, PO_4_ bound with Mg^2+^, we have searched Protein Data Bank and 5J33.pdb was used for our initial modeling of the first Mg^2+^ in the MTHFD2 binding pocket, in which MTHFD2 is modeled using 6KG2.pdb as template. Based on the knowledge we have gained from mutagenesis (D168A/E/N/S, S201R, D225A/N and R233A/K/S), we have manually added the second magnesium and then optimized on several levels: first we optimized the binding pocket (total 125 atoms) using 3-21G bases set and then we integrated the optimized binding pocket into the MTHFD2 complex. Several rounds of relaxation were carried out to let the system reach the equilibrium.

### Reference

1. Gustafsson R, Jemth A-S, Gustafsson NMS, Färnegårdh K, Loseva O, Wiita E, et al. Crystal Structure of the Emerging Cancer Target MTHFD2 in Complex with a Substrate-Based Inhibitor. Cancer Res. 2017;77: 937–948. doi:10.1158/0008-5472.CAN-16-1476
