# Supplementary material for: The catalytic mechanism of the mitochondrial methylenetetrahydrofolate dehydrogenase/cyclohydrolase (MTHFD2)
Source: PLoS Comput Biol. 2022 May 25;18(5):e1010140. doi: 10.1371/journal.pcbi.1010140 (PMC9173628; doi:10.1371/journal.pcbi.1010140)
Supplement: S2 Text — (DOCX) [file pcbi.1010140.s019.docx]

### Empirical valence bond (EVB) simulations

The empirical valence bond (EVB) approach is a well-developed and successfully used method [1,2]. It allows you to practically explore the catalytic landscape by calculating reaction free energies in a practical and accurate way. It represents the reacting system, ranging from reactions of molecules to catalysis in enzymes, in a realistic but simple way. The system usually is represented by different resonance forms (diabatic states) with specifically curated force fields. Hence, it provides quantitative comparison of the effect of different environments (particularly in solutions and in enzymes) on the reaction potential surfaces. The resonance forms, namely the reactant, intermediate, and product states, are described by both the general molecular mechanics (MMs) force field within Enzymix for the region that is not involved in the reaction and a quantum empirical force field to represent the reaction region (EVB atoms). The complete protocol of EVB methods have been described well elsewhere [1,3], here we focus on the detailed parameters we used in our work.

In addition, we have relaxed our systems for five rounds using MD simulation with 50 ns each at 300 K. There are total 9,502 protein atoms in our relaxing system: 2,321 atoms (324 residues) are in region II, and 7,181 are in region III. The center for water and region II is -64.47 8.96 -35.06 and the radius for region II is 18.0 Å. The RMSD of all protein heavy atoms of the last round is given in S5 Fig.

**References**

1. Kamerlin SCL, Warshel A. The EVB as a quantitative tool for formulating simulations and analyzing biological and chemical reactions. Faraday Discuss. 2010;145: 71–106. doi:10.1039/B907354J

2. King G, Warshel A. A surface constrained all‐atom solvent model for effective simulations of polar solutions. J Chem Phys. 1989;91: 3647–3661. doi:10.1063/1.456845

3. Lee FS, Chu ZT, Warshel A. Microscopic and semimicroscopic calculations of electrostatic energies in proteins by the POLARIS and ENZYMIX programs. J Comp Chem. 1993;14: 161–185. doi:https://doi.org/10.1002/jcc.540140205
