# Supplementary material for: The catalytic mechanism of the mitochondrial methylenetetrahydrofolate dehydrogenase/cyclohydrolase (MTHFD2)
Source: PLoS Comput Biol. 2022 May 25;18(5):e1010140. doi: 10.1371/journal.pcbi.1010140 (PMC9173628; doi:10.1371/journal.pcbi.1010140)
Supplement: S3 Text — (DOC) [file pcbi.1010140.s020.doc]

**Experimental evidence of the two Mg^2+^ system and its implications for catalysis**

Nicotinamide adenine dinucleotide (NAD^+^), as an essential and ubiquitous coenzyme, participates in fundamental and vital pathways including energy metabolism regulation, DNA damage repair and recombination, and post-translational modifications [1,2].

In order to identify crystallized structures of NAD^+^ bound with Mg^2+^, we have extracted 31,477 structures from Protein Data Bank by searching for ‘NAD’ and ‘Mg’ as ligands. Among these, 142 structures contain both NAD^+^ and Mg^2+^ which are listed in Table S4. After scrutinizing these structures, we decided to focus on the crystal structure of two proteins: (1) ketol-acid reductoisomerase (PDB ID: 4KQX), and (2) domain 1 of NAD^+^ riboswitch with nicotinamide adenine dinucleotide (NAD^+^) and the U1A protein (PDB ID: 7D7V, 7D7W and 7D81), the latter has two Mg^2+^ in close proximity of NAD^+^. However, NAD^+^ does not interact with protein in the riboswitch structures, namely 7D7V, 7D7W and 7D81 (see S11 Fig). Therefore, we will further investigate the structure of the ketol-acid reductoisomerase (PDB ID: 4KQX). The insights we gained from the structure of 4KQX (S10 Fig), indicated the existence of two Mg^2+^ ions, which further strengthen our concept of two Mg^2+^ binding modes in MTHFD2, corroborating previous data from direct mutagenesis and QM/MM studies. However, our free energy calculations indicated that one magnesium ion shows a slightly more energy favorable reaction kinetics but in terms of PO_4_ binding, the two magnesium system is more favorable.

Magnesium exists as Mg^2+^ ion in the living system and plays an important, sometimes essential role in metabolic pathways and nucleic acid biochemistry. Notably the DNA polymerase features two key magnesium ions that facilitate the addition of new nucleotides to a growing DNA chain [3]. Because we propose a two magnesium system from our modeling, our evaluation of the two magnesium system compared with a one/no magnesium system indicates that the presence of two magnesium significantly contribute to the PO_4_ binding but slightly slows down the reaction kinetics. Consistent with this, a study reported the pros and cons using two Mg^2+^ ions in the CDK2 reaction, where it can accelerate the reaction while slowing down the product release [4].

### **Reference**

1. Surjana D, Halliday GM, Damian DL. Role of Nicotinamide in DNA Damage, Mutagenesis, and DNA Repair. J Nucleic Acids. 2010;2010: 157591. doi:10.4061/2010/157591

2. Cantó C, Menzies KJ, Auwerx J. NAD+ Metabolism and the Control of Energy Homeostasis: A Balancing Act between Mitochondria and the Nucleus. Cell Metabolism. 2015;22: 31–53. doi:10.1016/j.cmet.2015.05.023

3. Yang L, Arora K, Beard WA, Wilson SH, Schlick T. Critical Role of Magnesium Ions in DNA Polymerase β’s Closing and Active Site Assembly. J Am Chem Soc. 2004;126: 8441–8453. doi:10.1021/ja049412o

4. Jacobsen DM, Bao Z-Q, O’Brien P, Brooks CL, Young MA. Price To Be Paid for Two-Metal Catalysis: Magnesium Ions That Accelerate Chemistry Unavoidably Limit Product Release from a Protein Kinase. J Am Chem Soc. 2012;134: 15357–15370. doi:10.1021/ja304419t
